# Report of a Case of Chronic Inflammation of the Vesiculae Seminales

**Published:** 1908-08

**Authors:** James A. Macleod

**Affiliations:** Buffalo, N. Y


### Report of a Case of Chronic Inflammation of the Vesiculae Seminales.

By JAMES A. MACLEOD, M.D. Buffalo, N. Y

THE vesiculæ seminales are liable to both acute and chronic inflammation. The inflammation is usually secondary in character, the infection taking place by simple extension either from the epididymis through the vas deferens or from the posterior urethra through the ejaculatory ducts.

The common causes are: (1) Gonorrhea; (2) Septic infection; (3) Tubercle.

Gonorrheal inflammation is generally secondary in character, the infection extending from the posterior urethra through the ejaculatory ducts; it is quite possible, however, that it may in a few cases be tertiary in character, the infection extending from a gonorrheal epididymitis, which was secondary to a urethral disease. It is very liable to become chronic and it is exceedingly difficult to affect a cure.

Septic inflammation is always secondary in character, the infection extending from a septic posterior urethritis through the ejaculatory ducts. Tubercular inflammation is usually secondary to tubercular disease of the epididymis or of the bladder, the infection extending through the vas deferens or ejaculatory ducts. Rarely is the disease primary.

The acute inflammations are treated by rest, by free action of the bowels, by the application of heat through the rectum and by the administration of salines and sedatives. The chronic inflammations are treated by tonics, by stripping the vesiculæ of their contents by digital massage through the rectum and by hot rectal douches.

Mr. "X" consulted me October 31, 1904, complaining of hemorrhage per urethra coming on during or after sexual excitement. He stated that he was quite healthy up to the year 1901, when he had, for the first time, a hemorrhage from the urethra. On that occasion he was sexually excited and the bleeding occurred unconsciously to him without pain. The hemorrhage recurred from time to time over a period of one and a half years. He was treated by a prominent genitourinary specialist for many months. His bladder was washed out two or three times a week, and he was forbidden sexual intercourse. No examination was made per rectum.

This patient stated that he was of a very passionate nature, working himself up, at times, to a high pitch of excitement and that up to the time of the first hemorrhage he had given free license to his physical passions. The hemorrhage did not however occur during or after sexual intercourse, but during or after conditions of intense sexual excitement in which coitus did not take place. A distended condition of the bladder was always associated with these conditions. He denied ever having had a urethritis, simple or specific.

His condition on consulting me may be best described by quoting my case notes as follows: The patient is a somewhat thin, fairly well nourished man but with a pale and pasty skin. He has a normal temperature and a pulse of 85 with good volume and tension. The tongue is clean, the appetite is good, the bowels are regular and not constipated. The physical signs in regard to the chest and the abdomen are nil. The testicles, epididymes and the vasa deferentia are not tender nor do they give any evidence of induration. The urethra and the bladder are normal to the sound. The urine is acid with a specific gravity of 1016, and has no albumin, pus or blood.

The rectal examination discloses that the sphincters ani are very tight; the prostate is not enlarged; the vesiculæ seminales are enlarged, tortuous, tender and stony hard. After gentle massage of the vesiculæ a mucus discharge was expressed through the urethra. The mucus was examined microscopically and demonstrated numerous pus cells, a few dead or nearly dead spermatozoa, a few blood corpuscles, but no microörganisms could be detected.

The blood was examined but nothing was demonstrated except a marked lowering of the hemoglobin percentage. I then sent him to Dr. J. Henry Dowd for cystoscopic examination, and he reported the bladder to be normal.

Mr. "X" was treated by tonics, by massage of the vesiculæ by the finger and recal staff, and by hot rectal saline douches. He was advised to avoid situations in which he might become sexually excited and he was advised not to allow his bladder to become over distended. These measures were continued up to January 15, 1905, when his condition was much improved; the vesiculæ were much softer, more easily stripped of their contents, but were still considerably indurated and much larger than normal. The strippings revealed nothing of pathological importance. The treatment was not kept up so rigidly thereafter, but examinations were made from time to time up to September 15, 1905, when the condition was in much the same state as at January 15, 1905.

He resumed sexual intercourse and again gave unbridled license to his physical passions up to September 1906, when he had a recurrence of the bleeding. Examination again revealed the vesiculæ enlarged but not tender, hard but not stony hard as they had been two years previous. The strippings were examined; blood and pus were demonstrated. Treatment similar in character to that used previously was again instituted. On November 23, 1906, Mr. "X" had a hemorrhage following a condition of intense sexual excitement and distended bladder. Thereafter the condition gradually improved until the vesiculæ became quite soft and easily stripped of their contents, which showed nothing of pathological importance.

On March 24, 1907, much to my surprise, a copious hemorrhage occurred following a condition of intense sexual excitement and full bladder. The question of operation arose, as it had a number of times before, but I decided to postpone operative measures until I had made an opsonic investigation. The strippings were examined and nothing of pathological importance was found, but on cultures being made a good growth of streptococci was obtained with a few isolated colonies of staphylococci; a bacillus was also noticed. The streptococci were exceedingly difficult to grow and to keep alive. The opsonic index to both the bacillus and the staphylococcus was normal but that to the streptococcus was extremely low, about .3. Separate vaccines were prepared from the staphylococcus and the streptococcus for the reason that it is necessary to give larger doses of the staphylococcus and also that it is necessary to give the streptococcus more frequently than the staphylococcus.

On May 7, 1907, I injected 20 million streptococci and 100 million staphylococci; twenty-four hours afterward, without warning, an exceedingly copious hemorrhage occurred. Thereafter I injected 10 million streptococci every other day and 100 million staphylococci twice a week, no other treatment being employed. His condition gradually improved, his weight increased, his hemoglobin percentage raised up to normal; his vesiculæ became soft, decreased in size and were easily stripped of their contents, which proved, on examination, to be normal in character. The index to the streptococcus was raised and maintained at a high level by the appropriate doses of the vaccine as mentioned above.

In conclusion, I would like to express the opinion that, in this case, there was a chronic ulceration of the vesiculæ seminales, involving not only the mucus membrane but also the submucus and muscular layers. How this ulceration arose I am not prepared to say but it was, undoubtedly, kept existent by the micro-örganisms discovered through the cultures made. The condition was aggravated by excessive sexual indulgence and the bleeding was caused by the rupture of small vessels in the bases of the ulcers. I would like to draw attention to the fact that, with the exception of the hemorrhage occurring after the first injection of the vaccines, the bleedings always took place during or immediately after a condition of intense sexual excitement associated with a distended bladder, but in which, however, intercourse did not take place. During such a state the bloodvessels in the region of the base of the bladder are greatly engorged, the blood pressure is high and the small vessels, mentioned above, are very liable to rupture. This gives a mechanical explanation to the actual bleeding and in my opinion such is the true explanation. In the case of the hemorrhage occurring after the first injection of the vaccines, the lowered vitality or resistance following an inoculation would explain the bleeding taking place without the usual raised condition of the blood pressure; but in addition I believe that there is a certain local congestion of the diseased part after an inoculation.

The anemia, demonstrated on several occasions by examination of the blood, was undoubtedly secondary in nature and improved as soon as the hemorrhages ceased to occur.

The treatment, especially the avoidance of conditions in which a high blood pressure existed, allowed the ulceration to improve and in fact to become quiescent but never becoming completely cured until the opsonic treatment was instituted. After the first inoculation all other treatment was stopped in order to give the procedure a fair and thorough trial. The general condition of the patient improved, pari passu, with the improvement of the vesicular disease.

327 Delaware Avenue.

